# Invisibility concentrator based on van der Waals semiconductor α-MoO_3_

**DOI:** 10.1515/nanoph-2021-0557

**Published:** 2021-11-30

**Authors:** Tao Hou, Sicen Tao, Haoran Mu, Qiaoliang Bao, Huanyang Chen

**Affiliations:** Department of Physics and Institute of Electromagnetics and Acoustics, Xiamen University, Xiamen 361005, China; Songshan Lake Materials Laboratory, Dongguan 523808, China; Shenzhen Exciton Science and Technology Ltd., Shenzhen 518052, P. R. China

**Keywords:** illusion effect, invisibility concentrator, van der Waals

## Abstract

By combining transformation optics and van der Waals layered materials, an invisibility concentrator with a thin layer of α-MoO_3_ wrapping around a cylinder is proposed. It inherits the effects of invisibility and energy concentration at Fabry–Pérot resonance frequencies, with tiny scattering. Due to the natural in-plane hyperbolicity in α-MoO_3_, the challenges of experimental complexity and infinite dielectric constant can be resolved perfectly. Through analytical calculation and numerical simulations, the relevant functionalities including invisibility, energy concentration and illusion effect of the designed device are confirmed, which provides guidelines for the subsequent experimental verification in future.

## 1 Introduction

Transformation optics [1, 2] has been a hot topic in recent decades. Based on the form invariance of Maxwell’s equations under coordinate transformation, it was proved that the optical path of light propagation in a continuous medium proves to be equivalent to that in a curved space after coordinate transformation. Hence, light behaviors can be manipulated at will through refractive index distribution of the material based on transformation optics. With the emergence and development of metamaterials, many transformation optical devices with different functions have been designed and implemented, such as invisibility cloaks [1], [2], [3], [4], carpet cloaks [5], [6], [7], [8], [9], [10], [11], optical illusion devices [12, 13], field rotators [14, 15], and so on. The refractive index distributions of transformation optical devices are usually complex, and the electromagnetic parameters are mostly inhomogeneous and anisotropic tensors, or even have some singularities [1, 2]. After perfect invisibility cloaks [2] were proposed for hiding objects from electromagnetic illumination, cylindrical versions [16] were also proposed theoretically for further fabrication. However, in order to achieve them more easily in experiments, it is usually necessary to simplify experimental parameters and avoid infinite electromagnetic parameters. One can use complex structures to equivalent gradient index materials to realize invisibility cloaks [3] and rotator [15] in microwaves. Of course, for all high-order cylindrical waves, although the simplified invisibility cloaks inherit some characteristics of the perfect cloaks, it has finite scattering [4]. According to the original invisibility mechanism, it was found that the Fabry–Pérot (F–P) resonance in extreme anisotropic materials can be used to design invisibility devices that worked well at multiple frequencies [17], where electromagnetic parameters are simplified [18] before long, and even applied to acoustic waves [19].

On the other hand, the hyperbolicity devices have been achieved using artificial materials (aka metamaterials), but expensive lithography techniques and complex manufacturing methods based on periodic subwavelength characteristics are involved [20], [21], [22], [23], [24]. In addition, the geometrical quality of the periodic cell and nonideal manufacturing process may affect the optical properties of the device [25]. The van der Waals (vdW) nanomaterials endowed by natural hyperbolicity [26], [27], [28], [29], [30], [31] have been reported with advantages in low price and the ease of processing [20, 32], which offer new possibilities for anisotropic dynamics and photonics. The phonon polaritons (PhPs) in polar vdW nanomaterials can be naturally endowed with hyperbolic response. However, most of these naturally occurring hyperbolic polaritons propagate out of the plane. Recently, α-MoO_3_ has been discovered as a biaxial van der Waals semiconductor material that can maintain a natural orthogonal in-plane phonon polarization mode in the infrared band [33], [34], [35]. Since α-MoO_3_ can support highly anisotropic PhPs and couple infrared (IR) light with anisotropic lattice vibrations, it provides an unprecedented platform for controlling energy flow, which has attracted much attention in recent years [36]. Unlike most hyperbolic materials, the three orthogonal crystal directions of α-MoO_3_ produce three different lattice modes, resulting in naturally in-plane hyperbolic anisotropy. Given the above findings, the dispersion relation of α-MoO_3_ flakes has been obtained by scattering scanning near-field optical microscopy (s-SNOM) and Fourier transform infrared (FTIR) spectroscopy [33], [34], [35], [36], [37], [38]. In the extra Reststrahlen band (RB) from 818 cm^−1^ to 974 cm^−1^, the real part of the permittivity of α-MoO_3_ is negative along the [100] direction and positive along the [001] direction. In the past, it was originally believed that extremely anisotropic electromagnetic properties (e.g., hyperbolicity) largely rely on the realization of artificial materials or transformation optic devices. But recent studies [27, 34, 39, 40] suggested that exotic optical behaviors may exist among the vast array of natural materials.

In this work, the invisibility and energy concentration characteristics are designed with new simplified parameters at the F–P resonance frequencies. The material parameters corresponding to the minimum scattering cross-section are obtained, in which the minimum scattering cross-section reaches 0.17(2*λ*/*π*). The simulation results show that when the cylindrical (rolled up) α-MoO_3_ materials are used to replace our metamaterials, the simplified invisibility concentrator can inherit the effects of electromagnetic invisibility and energy concentration of the perfect-invisibility devices [17], and the scattering cross-section reaches 0.24(2*λ*/*π*). Moreover, the invisibility concentrator of α-MoO_3_ has the illusion effect of source position when the point source is placed in particular regions.

## 2 Methods and theory

**Figure 1:**
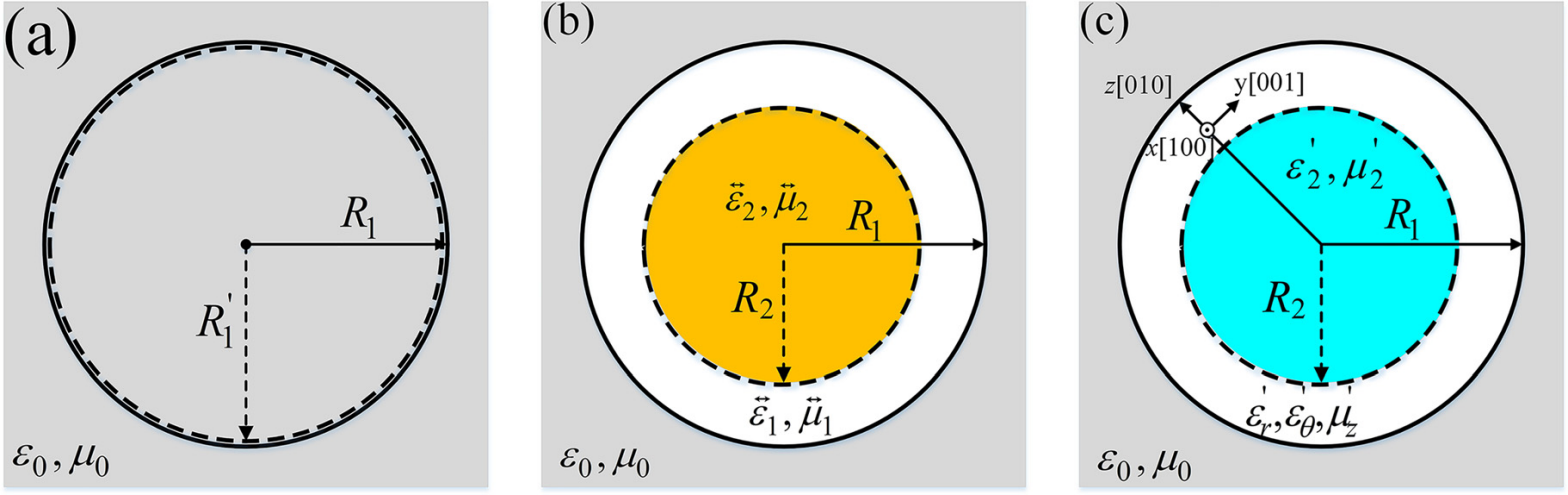
Perfect-invisibility concentrator. (a) Virtual space and (b) physical space of F–P transformation optics. (c) Physical space with simplified parameters.

In 2015, we proposed the perfect-invisibility based on transformation optics and F–P resonances [17], as shown in Figure 1(a) and (b).

By transforming coordinates in two dimensions

(1) $$\left\{ \begin{array}{c} r=r^{\prime }\left(r^{\prime }\geq R_{1}\right)\\ r=R_{2}+\frac{R_{1}-R_{2}}{R_{1}-R_{1}^{\prime }}\left(r^{\prime }-R_{1}^{\prime }\right)\left(R_{1}^{\prime }<r^{\prime }<R_{1}\right)\text{,}\\ r=\left(R_{2}/R_{1}^{\prime }\right)r^{\prime }\left(r^{\prime }\leq R_{1}^{\prime }\right) \end{array}\right.$$

the inner circle region of radius $R_{1}^{\prime }$ in virtual space can be compressed into the inner circle region of radius *R*_2_ in physical space, while the outer circle region of radius *R*_1_ keeps unchanged. After one dimensional singular mapping with $R_{1}=R_{1}^{\prime }$ , the materials in the region of *R*_2_ < *r* < *R*_1_ are called optical void media with extreme material parameters. *r* is the radius of physical space and $r^{\prime }$ is the radius of virtual space. We assume that virtual space is vacuum, and its dielectric constant and permeability are *ε*_0_ and *μ*_0_, respectively, while the permittivity and permeability tensors of the transformed materials in physics space are (in cylindrical coordinates):

(2) $$\left\{ \begin{array}{c} \frac{{\overset{↔}{\varepsilon }}_{1}}{\varepsilon_{0}}=\frac{{\overset{↔}{\mu }}_{1}}{\mu_{0}}=\left[\begin{array}{ccc} \frac{R_{1}-R_{2}}{R_{1}-R_{1}^{\prime }}\frac{r^{\prime }}{r} & 0 & 0\\ 0 & \frac{R_{1}-R_{1}^{\prime }}{R_{1}-R_{2}}\frac{r}{r^{\prime }} & 0\\ 0 & 0 & \frac{R_{1}-R_{1}^{\prime }}{R_{1}-R_{2}}\frac{r^{\prime }}{r} \end{array}\right]\left(R_{2}<r<R_{1}\right)\\ \frac{{\overset{↔}{\varepsilon }}_{2}}{\varepsilon_{0}}=\frac{{\overset{↔}{\mu }}_{2}}{\mu_{0}}=\left[\begin{array}{ccc} 1 & 0 & 0\\ 0 & 1 & 0\\ 0 & 0 & {\left(R_{1}^{\prime }/R_{2}\right)}^{2} \end{array}\right]\left(r<R_{2}\right) \end{array}\right.$$

When $R_{1}^{\prime }$ approaches to *R*_1_, Eq. (2) becomes

(3) $$\left\{ \begin{array}{c} \frac{{\overset{↔}{\varepsilon }}_{1}}{\varepsilon_{0}}=\frac{{\overset{↔}{\mu }}_{1}}{\mu_{0}}=\left[\begin{array}{ccc} \infty & 0 & 0\\ 0 & 0 & 0\\ 0 & 0 & 0 \end{array}\right]\left(R_{2}<r<R_{1}\right)\\ \frac{{\overset{↔}{\varepsilon }}_{2}}{\varepsilon_{0}}=\frac{{\overset{↔}{\mu }}_{2}}{\mu_{0}}=\left[\begin{array}{ccc} 1 & 0 & 0\\ 0 & 1 & 0\\ 0 & 0 & {\left(R_{1}/R_{2}\right)}^{2} \end{array}\right]\left(r<R_{2}\right) \end{array}\right.$$

In the region *R*_2_ < *r* < *R*_1_, since the effective refractive index in the *r* direction is zero, light propagates along such a path and does not accumulate any phase change. For the θ direction, the effective refractive index is infinite, which prevents light from propagating in this direction. Hence light can only propagate in the *r* direction. In the regions *r* > *R*_1_ and *r* < *R*_2_, the effective refractive index is 1 and *R*_1_/*R*_2_. Due to the functionality of the optical void mediums, the light passing through this ring structure does not produce any scattered and reflected energy, i.e., inducing invisibility. In addition, the field amplitude in the region *r* < *R*_2_ is significantly larger than that of the background medium, with a specific ratio of *R*_1_/*R*_2_, i.e., inducing energy concentration.

At this point, if we propose a similar cylindrical structure, as shown in Figure 1(c), when light propagates in transverse magnetic (TM) mode (*E_r_*, *E_θ_*, *H_z_*), the electromagnetic parameters of the cladding are redefined as ( $\varepsilon_{r}^{\prime }$ , $\varepsilon_{\theta }^{\prime }$ , $\mu_{z}^{\prime }$ ) = (∞, *ε*, 1), while the core material’s electromagnetic parameters are unchanged, i.e., ( $\varepsilon_{2}^{\prime }$ , $\mu_{2}^{\prime }$ ) = ((*R*_1_/*R*_2_)^2^, 1), where *ε* is constant. Now all electromagnetic parameters of such an invisibility concentrator are simplified as constants.

If the wavelength meets the F–P resonance condition in *r*-direction, i.e., the optical path is integer time of the wavelength [17],

(4) $$s=N\left(\frac{2\pi }{k_{0}}\right)=N\lambda \text{.}$$

with the optical path at this point as

(5) $$s=2\int_{R_{2}}^{R_{1}}n\text{d}l=2\sqrt{\varepsilon }\left(R_{1}-R_{2}\right)=N\left(\frac{2\pi }{k_{0}}\right)\text{.}$$

where “2” indicates that light rays pass through the concentrator twice. Then we can get the wave vector and wavelength of light transmission according to the electromagnetic parameters and the inner and outer radii.

When *r* < *R*_2_ and *r* > *R*_1_, the background material is isotropic. The magnetic field in these two regions can be expressed as the superposition of Bessel functions and Hankel functions of the first kind with

(6) $$H_{z}\left(r,\theta \right)=\left\{\begin{array}{l} \sum_{m=-\infty }^{\infty }\left[\alpha_{m}J_{m}\left(k_{0}r\right)+\beta_{m}H_{m}^{\left(1\right)}\left(k_{0}r\right)\right]{\text{e}}^{\text{i}m\theta }\left(r>R_{1}\right)\\ \sum_{m=-\infty }^{\infty }\gamma_{m}J_{m}\left(k_{0}R_{1}/R_{2}r\right){\text{e}}^{\text{i}m\theta }\left(r<R_{2}\right) \end{array}\right.\text{.}$$

where $\alpha_{m}$ , $\beta_{m}$ , and $\gamma_{m}$ are coefficients to be determined for each order of *m*.

For the region *R*_2_ ≤ *r* ≤ *R*_1_, the background material is anisotropic material, and the Maxwells’ equations are reduced as

(7) $$\frac{\partial }{\partial r}\left(\frac{r}{\varepsilon }\frac{\partial H_{z}}{\partial r}\right)+k_{0}^{2}rH_{z}=0.$$

The solution of Eq. (7) can be described as

(8) $$H_{z}\left(r\right)=c_{m}J_{0}\left(kr\right)+d_{m}H_{0}^{1}\left(kr\right)\text{,}$$

Among them, $J_{0}$ and $H_{0}^{1}$ are zero-order Bessel function and zero-order Hankel function of the first kind, respectively, *c*_*m*_ and *d*_*m*_ are coefficients to be determined for each order of *m*, (in this case, all the nonzero orders diminish), and $k=k_{0}\sqrt{\varepsilon }$ . Therefore, similar to Eq. (6), we rewrite Eq. (8) as the same series form of the magnetic field in this region

(9) $$H_{z}\left(r,\theta \right)=\sum_{m=-\infty }^{\infty }\left[c_{m}J_{0}\left(kr\right)+d_{m}H_{0}\left(kr\right)\right]{\text{e}}^{\text{i}m\theta },\;R_{2}\leq r\leq R_{1}\text{.}$$

In order to obtain the coefficients of the above expressions, we then explore the continuous boundary conditions of *H*_*z*_ and *E*_*θ*_ at the boundaries *r* = *R*_1_ and *r* = *R*_2_. Finally, we obtain four boundary equations according to Eqs. (6) and (9):

(10) $$\left\{ \begin{array}{c} \alpha_{m}J_{m}\left(k_{0}R_{1}\right)+\beta_{m}H_{m}^{1}\left(k_{0}R_{1}\right)=c_{m}J_{0}\left(kR_{1}\right)+d_{m}H_{0}^{1}\left(kR_{1}\right)\\ k_{0}\left[\alpha_{m}J_{m}^{\prime }\left(k_{0}R_{1}\right)+\beta_{m}H_{m}^{1^{\prime }}\left(k_{0}R_{1}\right)\right]=\frac{k}{\varepsilon }\left[c_{m}J_{1}\left(kR_{1}\right)+d_{m}H_{1}^{1}\left(kR_{1}\right)\right]\\ \gamma_{m}J_{m}\left(k_{0}R_{1}\right)=c_{m}J_{0}\left(kR_{2}\right)+d_{m}H_{0}^{1}\left(kR_{2}\right)]\\ k_{0}\frac{R_{2}}{R_{1}}\gamma_{m}J_{m}\left(k_{0}R_{1}\right)=\frac{k}{\varepsilon }\left[c_{m}J_{1}\left(kR_{2}\right)+d_{m}H_{1}^{1}\left(kR_{2}\right)\right] \end{array}\right.\text{.}$$

By substituting the Eqs. (5) and (10), we can explore all the coefficients and obtain the magnetic field of full form. Furthermore, the scattering cross-section of this invisibility concentrator is obtained as [41]

(11) $$\text{Scs}={\sum_{m=-\infty }^{\infty }\frac{2\lambda }{\pi }\left|\frac{\beta_{m}}{\alpha_{m}}\right|}^{2}\text{.}$$

## 3 Results and discussion

For the convenience of calculation and future experiment, the refractive index of the region *r* < *R*_2_ in Figure 1(c) is set as 1.4, which is considered as optical fiber in this article. The inner radius *R*_2_ is set as 10 μm and outer radius *R*_1_ is set as 14 μm (to keep the ratio of 1.4). *N* in Eq. (5) is set as 1. After analytically solving Eqs. (5) and (10), we find that when wavelength *λ* is set as 10.412 μm and the permittivity *ε* is set as 1.6938, the scattering cross-section reaches the minimum of 0.17(2*λ*/*π*), and the magnetic field amplitude in the region *r* < *R*_2_ is obviously greater than that in background region with the ratio of about 1.4. It shows that when we replace the original gradient permittivity material [17] with the constant permittivity material here, the invisibility and energy concentration can be achieved as well.

**Figure 2:**
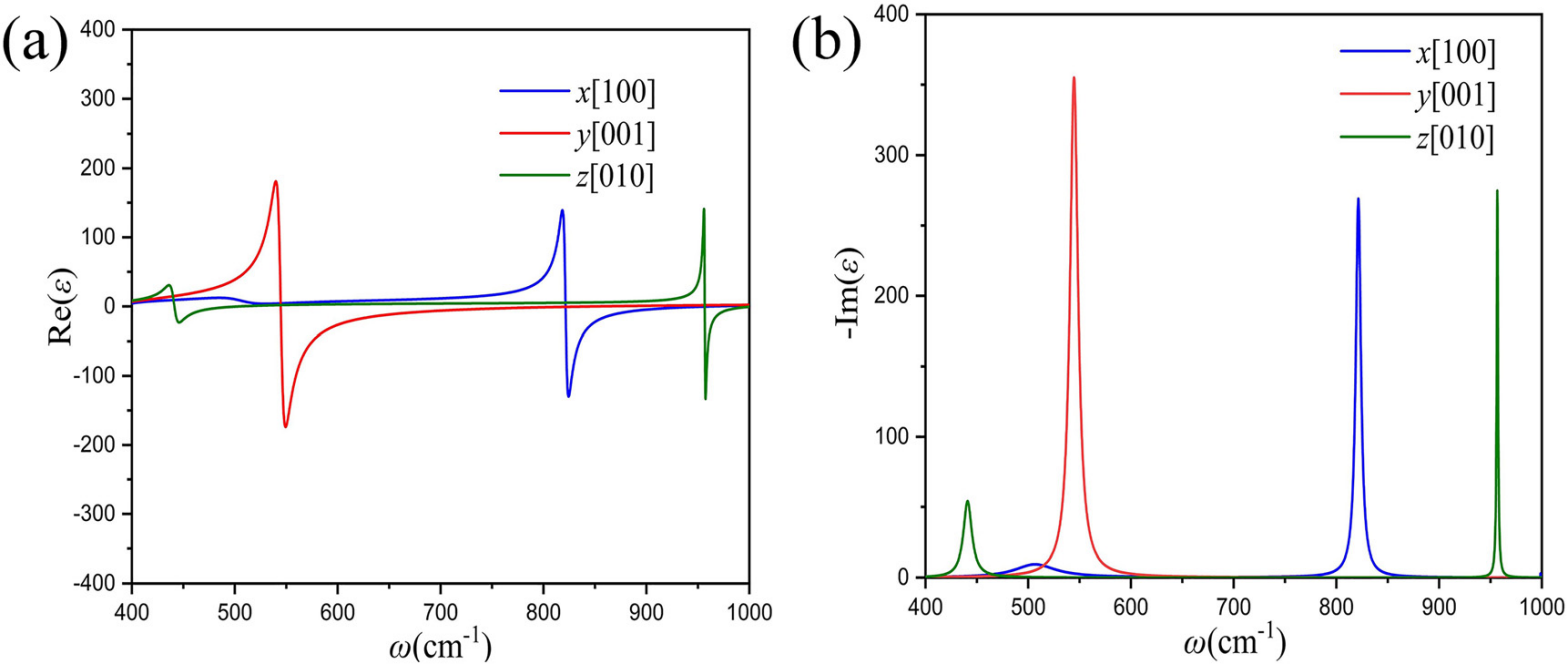
The relation between the (a) real and (b) imaginary part of the permittivity for α-MoO_3_ and the frequencies *ω*.

A natural question is what kind of material can be used to overcome the infinite permittivity and achieve the above proposed optical invisibility. Here we consider the natural planar hyperbolic polar van der Waals (vdW) nanomaterials α-MoO_3_. Through experimental measurement, the complex permittivity of α-MoO_3_ is obtained, as shown in Figure 2.

In Figure 1(c), if α-MoO_3_ are rolled on the inner core material, *x*[100] direction of α-MoO_3_ is set to be out of the plane and *z*[010] and *y*[001] directions correspond to the *r* and *θ* directions, respectively. When the incident wavelength is set as 10.461 μm (*ω* = 955.85 cm^−1^), the real part of the permittivity along *z*[010] direction reaches its maximum, and now the electromagnetic parameters are ( $\varepsilon_{r}^{\prime }$ , $\varepsilon_{\theta }^{\prime }$ , $\mu_{z}^{\prime }$ ) = (141.02 − 128.02*i*, 1.7322 − 0.057498*i*, 1). It is obvious that these parameters are very close to the best parameters discussed above. To check the invisibility and energy concentration effects based on α-MoO_3_, numerical simulations and analytical results are performed (the simulations below are all from commercial finite element solver COMSOL multiphysics).

**Figure 3:**
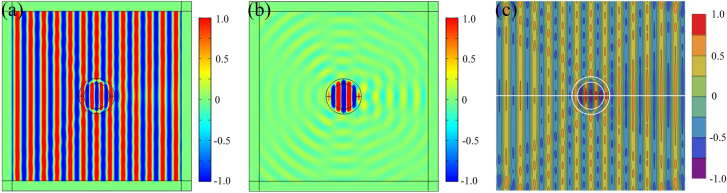
Numerical simulations of (a) *H*_*z*_ component diagram and (b) scattering magnetic field diagram based on α-MoO_3_ invisibility concentrator. (c) Analytical calculations of *H*_*z*_ component diagram based on α-MoO_3_ invisibility concentrator.

First, we adopted plane wave as an input source, and the results are shown in Figure 3.

According to Figure 3, although the cylinder is not perfectly invisible because the perfect invisibility requires perfect gradient materials and absolutely infinite permittivity, the structure has almost no external scattering when the plane wave incidents from left to right, which can also be proved by the tiny scattering cross-section of 0.24(2*λ*/*π*) that calculated from Eq. (11). In the region *r* < *R*_2_, the phase changes by about *π*/2 and the energy is strongly enhanced (from the magnetic amplitude diagram, not show here). The analytical solutions in Figure 3(c) are consistent with the numerical simulations in Figure 3(a), demonstrating the invisibility and energy concentration phenomenon.

**Figure 4:**
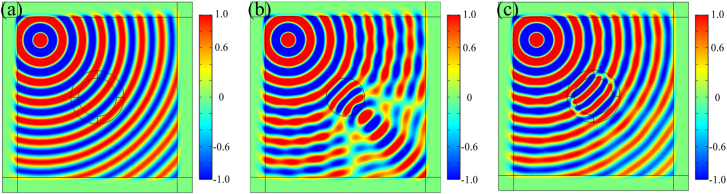
*H*_*z*_ component diagram in (a) vacuum, (b) pure optical fiber cylinder (without α-MoO_3_) and (c) α-MoO_3_ invisibility concentrator.

In order to further explore the invisibility characteristics of our structure, the incident wave is changed into a point source (which could be set by a tiny circle with a constant magnetic field) and it is placed at the upper left corner of the ring structure. The result is shown in Figure 4 (see Figure 4). Compared with the field diagram in vacuum, when the cylinder is only made up of optical fiber, light will propagate with an apparent scattering effect (or focusing effect) due to the higher refractive index of optical fiber. However, when the optical fiber rod is rolled up with α-MoO_3_, the magnetic field distribution outside the invisibility concentrator is similar to that in vacuum without any scattering. In addition, under the effects of the anisotropy of α-MoO_3_ and the F–P resonance, the phase change and energy enhancement still exist in this α-MoO_3_ invisibility concentrator.

**Figure 5:**
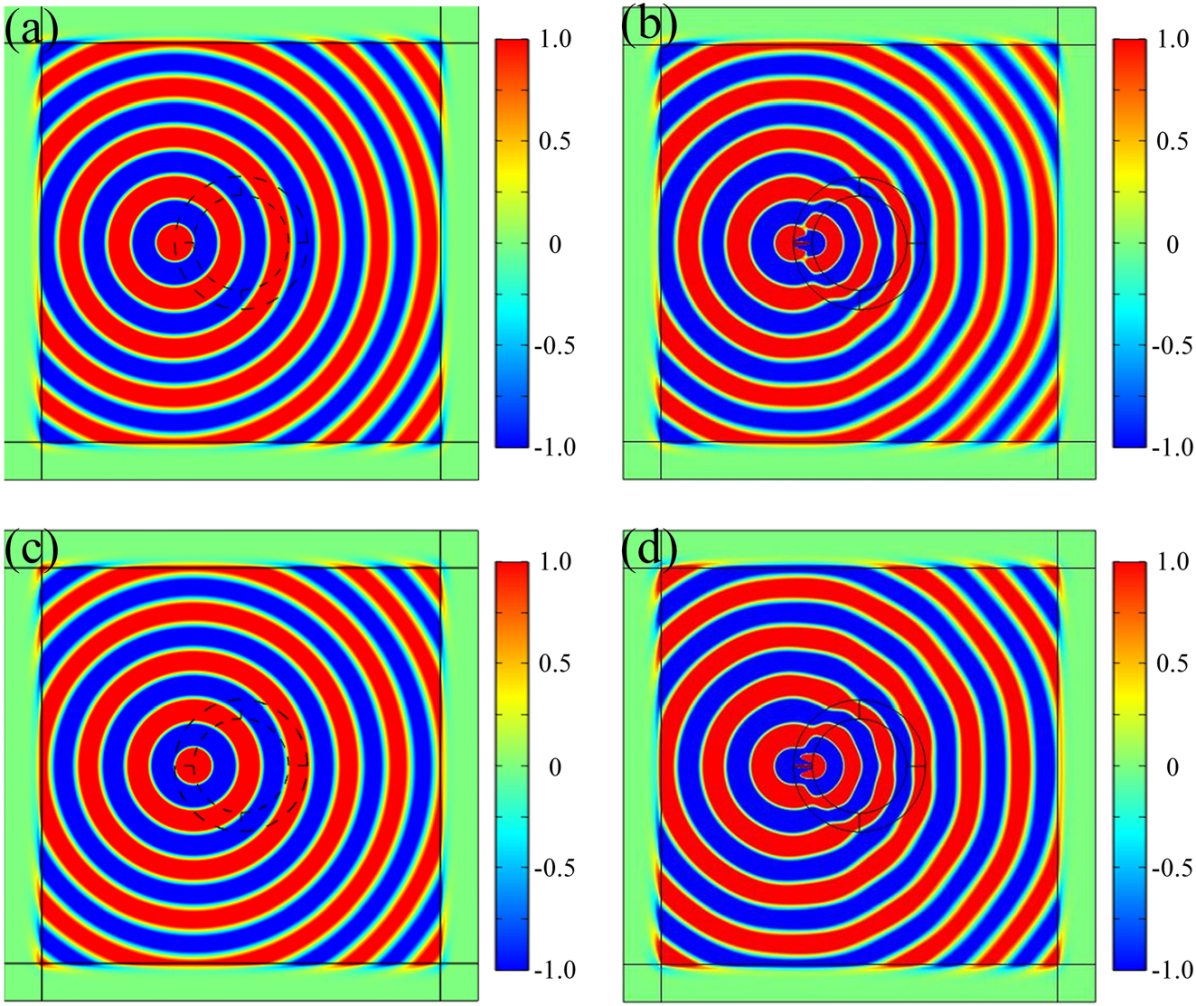
*H*_*z*_ component diagram when the point source is placed at *r* = *R*_1_ (a) in vacuum and (b) α-MoO_3_ invisibility concentrator and *r* = *R*_2_ in (c) vacuum and (d) α-MoO_3_ invisibility concentrator, respectively.

To explore the position illusion effect of invisibility concentrator, the point source is placed at *r* = *R*_1_ or *r* = *R*_2_, and the results are shown in Figure 5.

As shown in Figure 5, there are a few similarities and differences in the magnetic fields in vacuum and in α-MoO_3_ invisibility concentrator. Comparing Figure 5(a) and (b), the invisibility of α-MoO_3_ invisibility concentrator can be achieved when the point source is placed at *r* = *R*_1_, which is judged by the same field distribution outside the cylinder. In addition, when the point source is placed at the different position in α-MoO_3_ cladding (taking *r* = *R*_1_ and *r* = *R*_2_ as examples), the magnetic field in Figure 5(b) is the same as that in Figure 5(d) except for the difference of signs, which make it difficult for us to judge the specific position of the source by the magnetic field distribution of near field. As a contrast, by removing the α-MoO_3_ invisibility concentrator and placing the source at the same position of Figure 5(d), we obtain a new magnetic field as shown in Figure 5(c) and prove that it is the effect of α-MoO_3_ invisibility concentrator that leads to the difference between visual positions of sources in Figure 5(c) and (d). Hence, it can be concluded that people outside α-MoO_3_ invisibility concentrator only observe a point source placed at *r* = *R*_1_ when the point source is placed at any position in the region *R*_2_ < *r* < *R*_1_, i.e., the optical illusion effect of source position.

## 4 Conclusions

In this paper, a new invisibility concentrator based on α-MoO_3_ is proposed, which inherits invisibility and energy concentration from perfect-invisibility devices. The simulation results show that it can be invisible under both plane wave and point sources, which proves its omnidirectional invisibility. Moreover, it is found that when the point source is placed in a particular region of *R*_2_ < *r* < *R*_1_, the invisibility concentrator of α-MoO_3_ has the illusion effect of source position. Previous works [17, 18] showed that resonance phenomena still exist near the F–P resonance frequencies with slightly compromised effect. Therefore, such invisibility energy concentration effect based on α-MoO_3_ is not limited to a single frequency because the radial permittivities of α-MoO_3_ near all the polariton resonance frequencies are still extremely large while other permittivity components barely change. According to the different placing ways of α-MoO_3_ with the F–P resonance conditions, we can obtain the same effects in frequency bands near the other two polariton resonance frequencies in Figure 2(a). We suggest wrap a thin layer of α-MoO_3_ onto an optical fiber owing to the electrostatic effects and a 4 μm-thick α-MoO_3_ surrounding the cylinder could be achieved by wrapping a sufficiently wide α-MoO_3_ flake for a few tens circles with careful control of the rolling angle. Besides, there are other possible approaches. For example, high-quality α-MoO_3_ with large area can be directly grown on the SiO_2_ substrate by a CVD method [42]. Therefore, monocrystalline α-MoO_3_ film may be directly grown around the SiO_2_ cylinder by carefully controlling synthesis parameters. The results in this paper suggest that hyperbolic materials such as α-MoO_3_ and V_2_O_5_ could serve as a new basis for transformation optics, and give birth to many new nanophotonic concepts beyond invisibility concentrator, for example, multifrequency superscattering [43], ultrathin infrared polarizer [44], miniaturized hyperlenses [45], transformation polaritonics [46], improved infrared imaging [47] and detection system [48].
